# Advantages of pure platelet-rich plasma compared with leukocyte- and platelet-rich plasma in promoting repair of bone defects

**DOI:** 10.1186/s12967-016-0825-9

**Published:** 2016-03-15

**Authors:** Wenjing Yin, Xin Qi, Yuelei Zhang, Jiagen Sheng, Zhengliang Xu, Shicong Tao, Xuetao Xie, Xiaolin Li, Changqing Zhang

**Affiliations:** Department of Orthopaedic Surgery, Shanghai Jiao Tong University Affiliated Sixth People’s Hospital, Shanghai, China

**Keywords:** Platelet-rich plasma, Leukocyte- and platelet-rich plasma, Pure platelet-rich plasma, Bone regeneration, Animal model, Nuclear factor κB

## Abstract

**Background:**

High levels of pro-inflammatory cytokines in leukocyte- and platelet-rich plasma (L-PRP) may activate the nuclear factor κB (NF-κB) pathway to counter the beneficial effect of the growth factors on bone regeneration. However, to date, no relevant studies have substantiated this.

**Methods:**

L-PRP and pure platelet-rich plasma (P-PRP) were isolated. The in vitro effects of L-PRP and P-PRP on the proliferation, viability and migration of human bone marrow-derived mesenchymal stem cells (HBMSCs) and EaHy926, tube formation of EaHy926, and osteogenic differentiation of HBMSCs were assessed by cell counting, flow cytometry, scratch assay, tube formation assay, and real-time quantitative polymerase chain reaction (RT-PCR), western blotting and Alizarin red staining, respectively. The in vitro effects of L-PRP and P-PRP on the nuclear translocation of NF-κB p65, mRNA expression of inducible nitric oxide synthase and cyclooxygenase-2, and production of prostaglandin E2 and nitric oxid were assessed by western blotting, RT-PCR, enzyme-linked immunosorbent assay and Griess reaction, respectively. The in vivo effects of L-PRP or P-PRP preprocessed β-tricalcium phosphate (β-TCP) on the calvarial defects in rats were assessed by histological and immunofluorescence examinations.

**Results:**

P-PRP, which had similar platelet and growth factors concentrations but significantly lower concentrations of leukocytes and pro-inflammatory cytokines compared with L-PRP, promoted the proliferation, viability and migration of HBMSCs and EaHy926, tube formation of EaHy926 and osteogenic differentiation of HBMSCs in vitro, compared with L-PRP. The implantation of P-PRP preprocessed β-TCP also yielded better histological results than the implantation of L-PRP preprocessed β-TCP in vivo. Moreover, L-PRP treatment resulted in the activation of the NF-κB pathway in HBMSCs and EaHy926 in vitro while the postoperative delivery of caffeic acid phenethyl ester, an inhibitor of NF-κB activation, enhanced the histological results of the implantation of L-PRP preprocessed β-TCP in vivo.

**Conclusions:**

Leukocytes in L-PRP may activate the NF-κB pathway via the increased pro-inflammatory cytokines to induce the inferior effects on bone regeneration of L-PRP compared with P-PRP. Hence, P-PRP may be more suitable for bone regeneration compared with L-PRP, and the combined use of P-PRP and β-TCP represents a safe, simple, and effective alternative option for autogenous bone graft in the treatment of bone defects.

## Background

Challenges are still frequently encountered in the management of bone defects arising from trauma or pathology [1–3]. Although autogenous bone graft is still the gold standard, it suffers from inadequate tissue availability and associated donor site morbidity. Allografts and xenografts have been used as alternative options. However, there are concerns about the risks of disease transmission and rejection. To overcome these problems, β-tricalcium phosphate (β-TCP), a synthetic calcium phosphate ceramic, has been introduced because of the advantages of unlimited availability, consistent quality and good biocompatibility. β-TCP commonly functions as an osteoconductive material that lacks the osteogenic properties of autogenous bone, and therefore, the use of β-TCP alone in repairing critical size defects can be challenging [4, 5].

Platelet-rich plasma (PRP) is an autologous blood product composed of concentrated platelets. α-granules of concentrated platelets in PRP contain and release concentrated levels of platelet-derived growth factor (PDGF), transforming growth factor-β1 (TGF-β1), vascular endothelial growth factor (VEGF), and insulin-like growth factor (IGF) [6–8], which are known to have beneficial effects on osteogenesis [9–13]. Numerous studies have demonstrated that the use of autologous PRP in bone regeneration represents a safe, simple, and cost-efficient approach that has positive effects on cell proliferation and migration [14–16], osteogenesis [17–19], and angiogenesis [20, 21]. Consequently, the combined use of PRP and β-TCP has gained popularity in the field of bone tissue engineering for the combination of all properties required in an ideal bone graft material, including osteoinductivity, osteoconductivity, osteogenesis, and angiogenesis [22–24].

Despite the increasing use of PRP, the optimal PRP formulation for tissue regeneration is still unknown, and over the past few years attention has been drawn to the concentration of leukocytes in PRP. It has been demonstrated that high concentrations of leukocytes in PRP may deliver increased levels of pro-inflammatory cytokines, including interleukin-1β (IL-1β) and tumor necrosis factor-α (TNF-α), and result in the production of destructive proteases, together with inhibited formation and enhanced degradation of extracellular matrix [25–27]. IL-1β and TNF-α have been described to induce harmful effects on tissue regeneration through the activation of nuclear factor κB (NF-κB) signaling pathway. NF-κB heterodimers are normally located in the cytoplasm in an inactive form bound to inhibitory κB (IκB). Stimulation of receptive cells by IL-1β or TNF-α leads to the degradation of IκB to release NF-κB heterodimers and allow their subsequent translocation to the nucleus where they can activate the expression of a wide range of regulatory genes involved in apoptosis, inflammation, and other immune responses [28]. Therefore, leukocyte- and platelet-rich plasma (L-PRP) may activate the NF-κB pathway via IL-1β and TNF-α to induce deleterious effects on tissue regeneration. However, to date, no relevant studies have substantiated this.

Considering the harmful effects of pro-inflammatory cytokines released from leukocytes on tissue regeneration, effort has been put into the depletion of the leukocytes in PRP to prepare pure platelet-rich plasma (P-PRP), in an attempt to achieve maximum therapeutic benefit [25, 29]. While the superior effects of P-PRP over L-PRP have been proved on regeneration of articular cartilage [26, 30–32] and tendons [33–36], they have not yet been evaluated on bone regeneration.

The objective of the current study is to evaluate the effects of L-PRP and P-PRP on bone regeneration and the NF-κB pathway in vitro and in vivo, in order to develop an alternative method for autogenous bone graft.

## Methods

This study was in adherence with the Declaration of Helsinki. Independent Ethics Committee and the Animal Care and Use Committee of Shanghai Jiao Tong University Affiliated Sixth People’s Hospital approved the protocols of this study (No. 2015-33 and No. DWSY2014-33). Written informed consent was obtained from each human volunteer.

### Preparation and analysis of human PRPs

#### Preparation of human PRPs

Whole blood was collected from ten healthy human volunteers (6 men and 4 women, 21–45 years old) into acid-citrate dextrose solution A (ACD-A) anticoagulant (1 mL ACD-A/9 mL blood). Each blood sample was divided and used to prepare L-PRP and P-PRP in a single-donor model.

L-PRP was prepared using WEGO PRP preparation system (WEGO, Weihai, Shandong, China), the only commercial PRP preparation system approved by the China Food and Drug Administration. P-PRP was prepared using a method developed in our laboratory, which was demonstrated to be able to concentrate platelets similarly to WEGO PRP preparation system while removing leukocytes and erythrocytes (data unpublished). To prepare P-PRP using the method developed in our laboratory, 40 mL of whole blood was spun at 160*g* for 10 min in a 50-mL centrifuge tube to separate platelet-containing plasma from erythrocytes and leukocytes, and then the separated plasma was transferred to a new tube and spun again at 250*g* for 15 min. After discarding supernatant plasma, precipitated platelets were resuspended in the residual plasma to obtain 4 mL of P-PRP.

#### Quantification of leukocyte and platelet concentrations

Leukocyte and platelet concentrations of whole blood, L-PRP, and P-PRP were determined by whole blood analysis using an automatic hematology analyzer (XS-800i, Sysmex, Kobe, Japan).

#### Quantification of growth factors and pro-inflammatory cytokine concentrations

PDGF-AB, TGF-β1, VEGF, IL-1β, and TNF-α concentrations of whole blood, L-PRP, and P-PRP were quantified by enzyme-linked immunosorbent assay (ELISA) [30]. In brief, whole blood, L-PRP, and P-PRP were incubated with 10 % CaCl_2_ (final concentration 22.8 mM) at 37 °C for 7 days. At the end of the incubation period, the formulations were spun at 2800*g* for 15 min, and the supernatants were collected and assayed for growth factors and pro-inflammatory cytokine concentrations using the Quantikine Human Immunoassay kits (R&D Systems, Minneapolis, MN, USA) according to manufacturer’s instructions.

### Evaluation of the effects of L-PRP and P-PRP on cells in vitro

#### Isolation and expansion of cells

Human bone marrow-derived mesenchymal stem cells (HBMSCs) were isolated according to the protocols described previously [37]. In brief, bone marrow aspirates were harvested from the greater trochanter of 14 femur fractures patients (8 men and 6 women, 25–48 years old) during surgery. Bone debris were removed by filtering and cells were then cultured in 75 cm^2^ flasks at a density of 5.0 × 10^5^ cells/flask in the α-modification of minimum essential medium (α-MEM; Sigma-Aldrich, St Louis, MO, USA) containing 10 % fetal bovine serum (FBS; Gibco, Carlsbad, CA, USA) and 1 % antibiotics (penicillin G and streptomycin, Gibco, Carlsbad, CA, USA) at 37 °C in a humidified atmosphere containing 5 % CO_2_. The medium was changed after 48 h to remove non-adherent cells and thereafter every 3 days. Cells were detached with 0.25 % trypsin–EDTA (Invitrogen, Rockford, IL, USA) and passaged at ~80 % confluence. Cells at the fifth passage were used for this study.

EaHy926, the human umbilical vein endothelial cell line, was purchased from the American Type Culture Collection (Manassas, VA, USA) and cultured in Dulbecco’s Modified Eagle’s Medium (DMEM; Sigma-Aldrich, St Louis, MO, USA) containing 10 % FBS and 1 % antibiotics at 37 °C in a humidified atmosphere containing 5 % CO_2_. Cells were passaged at ~80 % confluence.

#### Cell proliferation analysis

Cells were seeded in 96-well plates at a density of 4000 cells/well and cultured for 24 h in serum-free medium to permit them to adhere. Cells were then cultured in medium supplemented with 10 % (v/v) of FBS, L-PRP, or P-PRP for 7 days. 10 % was selected because that it is the most frequently used PRP concentration in in vitro studies, and may be comparable to the concentration of PRP reached during in vivo administration [15]. Cell proliferation was assessed on days 1, 3, 5, and 7 using Cell Counting Kit-8 (CCK-8; Dojindo, Kumamoto, Japan) according to manufacturer’s instructions.

#### Cell viability and apoptosis analysis

Cells were seeded in 6-well plates at a density of 1.0 × 10^5^ cells/well, serum-starved for 24 h, and treated with 10 % of FBS, L-PRP, or P-PRP for 7 days. The cells were then incubated with 10 μM camptothecin (MedChem Express, Monmouth Junction, NJ, USA) for 6 h to induce apoptosis. Cell viability and apoptosis was analyzed by staining with Annexin V and PI [37]. Briefly, cultured cells were detached, centrifuged, resuspended in phosphate buffered saline (PBS; Gibco, Carlsbad, CA, USA) and stained with an Annexin V-FITC/PI staining kit (Cell Signaling Technology, Denvers, MA, USA). Data was acquired and analyzed by flow cytometry and guavaSoft (Guava easyCyte 8HT flow cytometry system, Millipore, MA, USA).

#### Cell migration analysis

Cells were seeded at a density of 2.1 × 10^4^ cells/well in Culture-Inserts (ibidi, Martinsried, Germany) and serum-starved as above. After removing the inserts to create a cell-free gap of 500 μm, cells were treated with 10 % of FBS, L-PRP, or P-PRP for 24 h. Cell migration was observed using an inverted microscope (Leica, Heidelberg, Germany) and quantified using WimScratch software (Wimasis, Munich, Germany) after 0, 12, and 24 h.

#### Tube formation analysis

Confluent EaHy926 were serum-starved for 24 h, detached, seeded at the density of 1.0 × 10^4^ cells/well in μ-slide angiogenesis plates (ibidi, Martinsried, Germany) precoated with 10 μL/well Matrigel (BD, Oxford, UK) and treated with DMEM containing 10 % of FBS, L-PRP, or P-PRP for 24 h. Tube formation was observed using an inverted microscope and quantified using WimTube software (Wimasis, Munich, Germany) after 12 and 24 h.

#### Osteogenic differentiation induction of HBMSCs

For osteogenic differentiation induction, nearly confluent HBMSCs were treated with osteogenic differentiation medium containing 10 % of FBS, L-PRP, or P-PRP. The osteogenic differentiation medium was α-MEM supplemented with 10^−2^ M β-sodium glycerophosphate (Sigma-Aldrich, St Louis, MO, USA), 50 μg/mL L-ascorbic acid (Sigma-Aldrich, St Louis, MO, USA), and 1 % antibiotics. The medium was changed every 3 days.

#### Real-time quantitative polymerase chain reaction

After osteogenic differentiation induction for 14 days, real-time quantitative polymerase chain reaction (RT-PCR) was performed to detect the levels of mRNA expression of runt-related transcription factor 2 (Runx2; an early osteogenic marker) and osteocalcin (OC; a late osteogenic marker). Total RNA was extracted from cultured HBMSCs using TRIzol reagent (Invitrogen, Rockford, IL, USA). Reverse transcription was performed using the High Capacity Reverse Transcription kit (Invitrogen, Rockford, IL, USA). RT-PCR was then performed using SYBR Green detection reagent (SYBR Premix, Roche, Stockholm, Sweden). β-Actin was used as a housekeeping gene for the normalization of data. The primer sequences for the target genes are listed in Table 1. Data were analyzed using the ^ΔΔ^Ct method as described previously [38].

**Table 1:** The primers sequences used for RT-PCR

	Forward primer sequence (5′–3′)	Reverse primer sequence (5′–3′)
Runx2	CCAACCCACGAATGCACTATC	TAGTGAGTGGTGGCGGACATAC
OC	CCCCCTCTAGCCTAGGACC	ACCAGGTAATGCCAGTTTGC
COX-2	CTTCACGCATCAGTTTTTCAAG	TCACCGTAAATATGATTTAAGTCCAC
iNOS	GCTGCCAAGCTGAAATTGA	GATAGCGCTTCTGGCTCTTG
β-Actin	TTCAACACCCCAGCCATGT	GTGGTACGACCAGAGGCATACA

#### Western blotting

Protein expression of Runx2 in HBMSCs was analyzed by western blotting after osteogenic differentiation induction for 14 days. HBMSCs were lysed using mammalian protein extraction reagent (Pierce, Rockford, IL, USA) supplemented with complete protease inhibitor, and the total protein concentration was detected using BCA Protein Assay Kit (Pierce, Rockford, IL, USA). SDS-PAGE was performed using 100 μg of total protein and then transferred to a PVDF (Millipore, Billerica, MA, USA). After blocking with a solution of low-fat milk protein, the membranes were incubated with anti-Runx2 antibody (abcam, Cambridge, MA, USA) and anti-GAPDH (Cell Signaling Technologies, Danvers, MA, USA), followed by peroxidase-conjugated secondary antibodies. The blots were subjected to chemiluminescence detection using ECL western blotting substrate (Pierce, Rockford, IL, USA).

#### Analysis of osteocalcin production

To avoid artifacts due to the presence of OC in blood products [39], the medium above HBMSCs was changed into serum-free α-MEM after osteogenic differentiation induction for 14 days. After incubation for 24 h, the conditioned medium above HBMSCs was collected to quantify the amount of OC released by ELISA using Quantikine Human Osteocalcin Immunoassay kit (R&D Systems, Minneapolis, MN, USA).

#### Alizarin red staining

After osteogenic differentiation induction for 21 days, alizarin red staining was performed to visualize the extracellular accumulation of calcium [40]. In brief, cultured cells were fixed 4 % paraformaldehyde for 30 min, stained with 2 % alizarin red (Sigma-Aldrich, St Louis, MO, USA) for 30 min, rinsed with PBS for three times. After observing the mineral nodules using an inverted microscope, the cultures were incubated with 20 % methanol/10 % acetic acid solution (Sinopharm Chemical Reagent, Shanghai, China) for 15 min followed by measurement of the absorbance value at 450 nm.

### Evaluation of the effects of PRPs on NF-κB pathway in vitro

#### Cell culture conditions

Nearly confluent cells were serum-starve for 24 h and treated with medium supplemented with 10 % of FBS, L-PRP, or P-PRP. The cells were collected after 1 hour for western blotting or after 24 h for RT-PCR. For production of prostaglandin E2 (PGE2) and nitric oxide (NO) analysis, the medium was changed into serum-free medium after incubation for 24 h to minimize the potential artifacts due to the presence of PGE2 and NO in blood products. After 24 h, the conditioned medium was collected for the analyses.

#### Western blotting

Expression of NF-κB p65 (a subunit of NF-κB heterodimers) in the nucleus was analyzed by western blotting. Nuclear protein extracts were prepared using NE-PER Nuclear and Cytoplasmic Extraction Reagents Kit (Thermo Fisher Scientific, Rockford, IL, USA) according to manufacturer’s instructions. Western blotting was then performed as above using anti-NF-κB p65 antibody (Cell Signaling Technologies, Danvers, MA, USA).

#### RT-PCR

mRNA expression of inducible nitric oxide synthase (iNOS) and cyclooxygenase-2 (COX-2) were analyzed by RT-PCR, as above. The primer sequences for the target genes are listed in Table 1.

#### Analysis of PGE2 and NO production

The amount of PGE2 released into the medium was quantified by ELISA using the Quantikine Human Immunoassay kits (R&D Systems, Minneapolis, MN, USA) according to manufacturer’s instructions. The production of NO was measured by Griess reaction using the NO Assay Kit (Thermo Fisher Scientific, Rockford, IL, USA) according to manufacturer’s instructions.

### Evaluation of the effects of PRPs on bone regeneration in vivo

#### Animals

Forty male Sprague–Dawley rats (12 weeks old, weight 250–300 g) were used to create a calvarial defects model in this study and divided into four groups: (1) control (n = 10): unpreprocessed β-TCP was used to treat the defects; (2) L-PRP group (n = 10): autologous L-PRP was used to preprocess the β-TCP implanted; (3) L-PRP + caffeic acid phenethyl ester (CAPE) group (n = 10): CAPE, a specific inhibitor of NF-κB activation [41], was delivered to rats treated with L-PRP preprocessed β-TCP postoperatively; (4) P-PRP group (n = 10): autologous P-PRP was used to preprocess the β-TCP used.

#### Preparation of rat PRPs and transplanted constructs

Autologous whole blood was collected from each rat into ACD-A according to the protocols mentioned previously [42]. An equal volume of sterile saline was immediately injected to replace the collected blood. 0.15 mL of L-PRP or P-PRP was prepared as described. Platelet and leukocyte concentrations of whole blood, L-PRP, and P-PRP of were quantified by whole blood analysis. Sterile β-TCP scaffolds were then immersed in autologous L-PRP or P-PRP at 4 °C overnight to prepare transplanted constructs.

#### Animal surgery

The calvarial defects model in rats was created as reported previously [43]. Briefly, after anesthetization was achieved, a 1.0 cm sagittal incision was made on the scalp to expose the calvarium. A full-thickness defect 5 mm in diameter was created in the central area of each parietal bone using a trephine bur. The bone defects were then treated with prepared transplant constructs.

Each rat received an intramuscular injection of penicillin G (SPH, Shanghai, China) at a dose of 160,000 U/kg postoperatively. All rats had ad libitum access to food and water. Rats in the L-PRP + CAPE group received 10 μmol/kg/day CAPE intraperitoneally to inhibit the activation of NF-κB [44]. All rats were euthanized at 8 weeks postoperatively to harvest calvarias.

#### Micro-computed tomography scanning

Harvested calvarias were fixed in 4 % paraformaldehyde in 0.1 M phosphate buffer (pH 7.2) for 72 h and scanned using micro-computed tomography (micro-CT; Skyscan 1076, Kontich, Belgium) to evaluate new bone formation in the defects [43]. Scanning was performed at a resolution of 18 μm and the image data was used to analyze local bone mineral density (BMD) and bone volume to total bone volume (BV/TV) of the regenerated bone by NRecon software (Skyscan, Kontich, Belgium).

#### Sequential fluorescent labeling

Polychrome sequential fluorescent labeling was performed according to the protocols described previously to observe the amount of new bone formation and mineralization [43]. Briefly, rats received an intraperitoneal injection of 25 mg/kg tetracycline (Sigma-Aldrich, St Louis, MO, USA), 30 mg/kg alizarin red (Sigma-Aldrich, St Louis, MO, USA) and 20 mg/kg calcein (Sigma-Aldrich, St Louis, MO, USA) at 2, 4, and 6 weeks postoperatively, respectively. The calvarias were harvested at 8 weeks postoperatively, fixed in 4 % paraformaldehyde solution, dehydrated in a graded series of ethanol, embedded in methyl methacrylate, and sectioned into 150 μm thick specimens in the orientation of the sagittal surface. The sections were then glued onto a plastic support to polish a final thickness of approximately 50 μm. The sections were then observed for fluorescent labeling using a confocal laser scanning microscope (Leica, Heidelberg, Germany). The area of the newly formed bone was measured using Image Pro Plus (Media Cybernetics, Silver Springs, MD, USA) and expressed as a fraction of the total defect area.

#### Histological and immunohistochemical analysis

Harvested calvarias were decalcified in 10 % EDTA for 14 days, dehydrated with graded ethanol solutions, embedded in paraffin and sectioned at 5 μm at the central area of the defect. Sections were stained with hematoxylin and eosin (HE) to observe new bone formation. The area of the regenerated bone was measured using Image Pro Plus and expressed as a fraction of the total defect area.

The expression and distribution of OC and CD31 were evaluated by immunohistochemical staining using the primary antibodies (Abcam, Cambridge, MA, USA) at 4 °C overnight followed by HRP-conjugated anti-rabbit IgG for 1 h at 37 °C. Staining was developed in diaminobenzidine solution, with hematoxylin counterstaining. The number of blood vessels, which were defined by CD31-positive staining and a typical round or oval structure, was measured using Image Pro Plus.

### Statistical analysis

Data were analyzed using the Statistical Package for Social Sciences version 22.0 (SPSS, Chicago, IL, USA) and presented as mean ± standard deviation (SD). One-way analysis of variance (ANOVA) and Bonferroni post hoc test were performed for statistical analysis. Pearson correlation analysis was conducted to analyze the linear correlations between blood cells concentrations and cytokines concentrations. A *P* value less than 0.05 was considered statistically significant.

## Results

### Components of human whole blood and PRPs

**Fig. 1:**
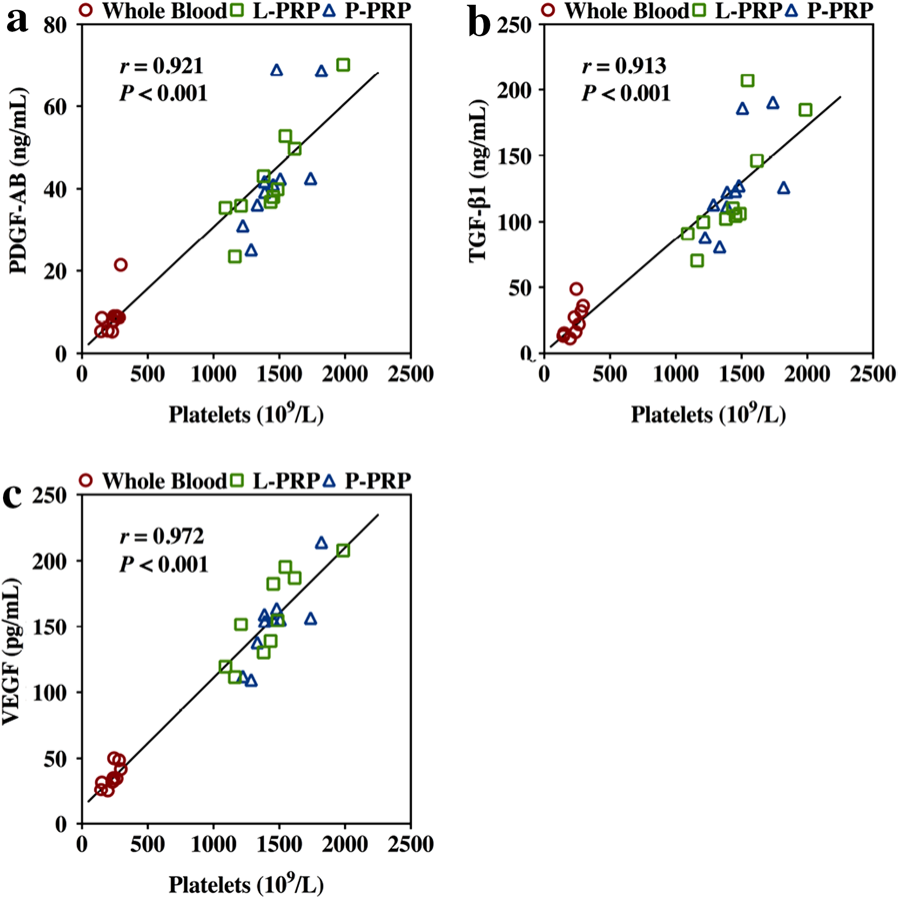
Correlations between platelet concentration and PDGF-AB concentration (**a**), TGF-β1 concentration (**b**), and VEGF concentration (**c**)

The results demonstrated that platelets, PDGF-AB, TGF-β1, and VEGF were significantly concentrated in L-PRP and P-PRP compared with the whole blood, and there was no significant difference between L-PRP and P-PRP (Table 2). There were significantly positive correlations between platelet concentration and PDGF-AB concentration (*r* = 0.921, *P* < 0.001, Fig. 1a), and TGF-β1 concentration (*r* = 0.913, *P* < 0.001, Fig. 1b), and VEGF concentration (*r* = 0.972, *P* < 0.001, Fig. 1c).

**Table 2:** Components of human whole blood and PRPs

	Platelet concentration (10^9^/L)	Leukocyte concentration (10^9^/L)	PDGF-AB concentration (ng/mL)	TGF-β1 concentration (ng/mL)	VEGF concentration (pg/mL)	IL-1β concentration (pg/mL)	TNF-α concentration (pg/mL)
WB	228.30 ± 50.50	6.18 ± 1.57	8.87 ± 4.67	23.83 ± 12.07	35.92 ± 8.51	11.68 ± 5.90	9.31 ± 5.05
L-PRP	1436.70 ± 257.97	34.58 ± 8.48	42.54 ± 12.65	122.22 ± 43.42	157.89 ± 33.64	93.33 ± 69.88	54.67 ± 27.10
P-PRP	1461.80 ± 189.14	0.18 ± 0.18	43.71 ± 14.39	126.95 ± 35.96	151.82 ± 29.29	3.83 ± 2.61	2.20 ± 0.78
Comparison, *P* value							
ANOVA	<0.001*	<0.001*	<0.001*	<0.001*	<0.001*	<0.001*	<0.001*
WB vs. L-PRP	<0.001*	<0.001*	<0.001*	<0.001*	<0.001*	<0.001*	<0.001*
WB vs. P-PRP	<0.001*	0.036*	<0.001*	<0.001*	<0.001*	>0.999	0.979
L-PRP vs. P-PRP	>0.999	<0.001*	>0.999	>0.999	>0.999	<0.001*	<0.001*

The asterisks indicate the significant differences between groups on ANOVA and with use of the Bonferroni post hoc test for multiple comparisons (*P* < 0.05)

*WB* whole blood

**Fig. 2:**
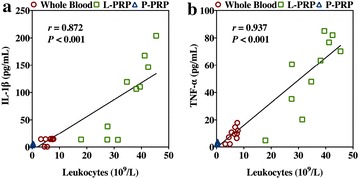
Correlations between leukocyte concentration and IL-1β concentration (**a**), and TNF-α concentration (**b**)

The results also demonstrated that the leukocyte concentrations were significantly different for all formulations, with L-PRP as being the highest concentration and P-PRP as being the lowest concentration (Table 2). Similarly, L-PRP also had the highest IL-1β and TNF-α concentrations compared with other formulations (Table 2). Although P-PRP had lower IL-1β and TNF-α concentrations compared with the whole blood, the differences of those between P-PRP and the whole blood were not significant (*P* > 0.999, and *P* = 0.979, respectively). There were significantly positive correlations between leukocyte concentration and IL-1β concentration (*r* = 0.872, *P* < 0.001, Fig. 2a), and TNF-α (*r* = 0.937, *P* < 0.001, Fig. 2b).

### P-PRP promotes the proliferation, viability and migration of HBMSCs and EaHy926 more effectively than L-PRP

**Fig. 3:**
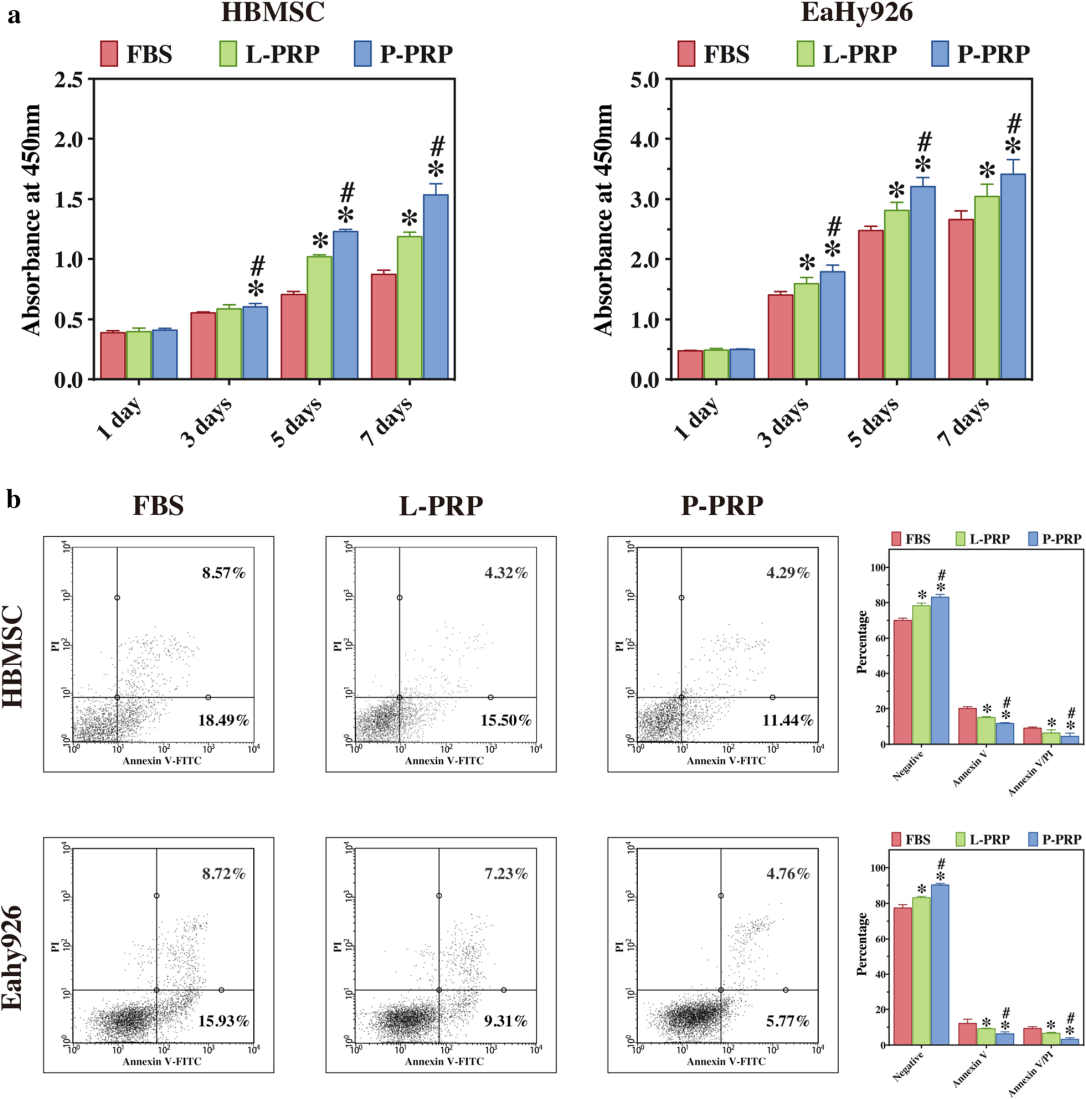
Effects of PRPs on the proliferation and viability of HBMSCs and EaHy926. **a** cell proliferation was analyzed with CCK-8 assay; **b** cell viability was analyzed with Annexin V/PI staining and flow cytometry quantification. Compared with FBS, both L-PRP and P-PRP promoted the proliferation and viability of HBMSCs and EaHy926, with P-PRP showing greater effects. *Bars* represent the means and standard deviation (n = 5); * indicates the statistically significant difference between PRPs and FBS (*P* < 0.05); ^#^ indicates the statistically significant difference between P-PRP and L-PRP (*P* < 0.05)

As shown in Fig. 3a, HBMSCs and EaHy926 proliferated gradually with increase in the culture time for all groups, whereas the proliferation of HBMSCs and EaHy926 was substantially promoted in the presence of PRP, with the highest proliferation observed in the presence of P-PRP.

The results of Annexin V/PI apoptosis assay are shown in Fig. 3b. The percentages of negative, Annexin V positive, and Annexin V/PI double positive cells represent the percentages of viable cells, early apoptotic cells, and late apoptotic or dead cells, respectively. The results demonstrated that both L-PRP and P-PRP inhibited the camptothecin-induced apoptosis to enhance the viability of HBMSCs and EaHy926, with P-PRP showing greater effects.

**Fig. 4:**
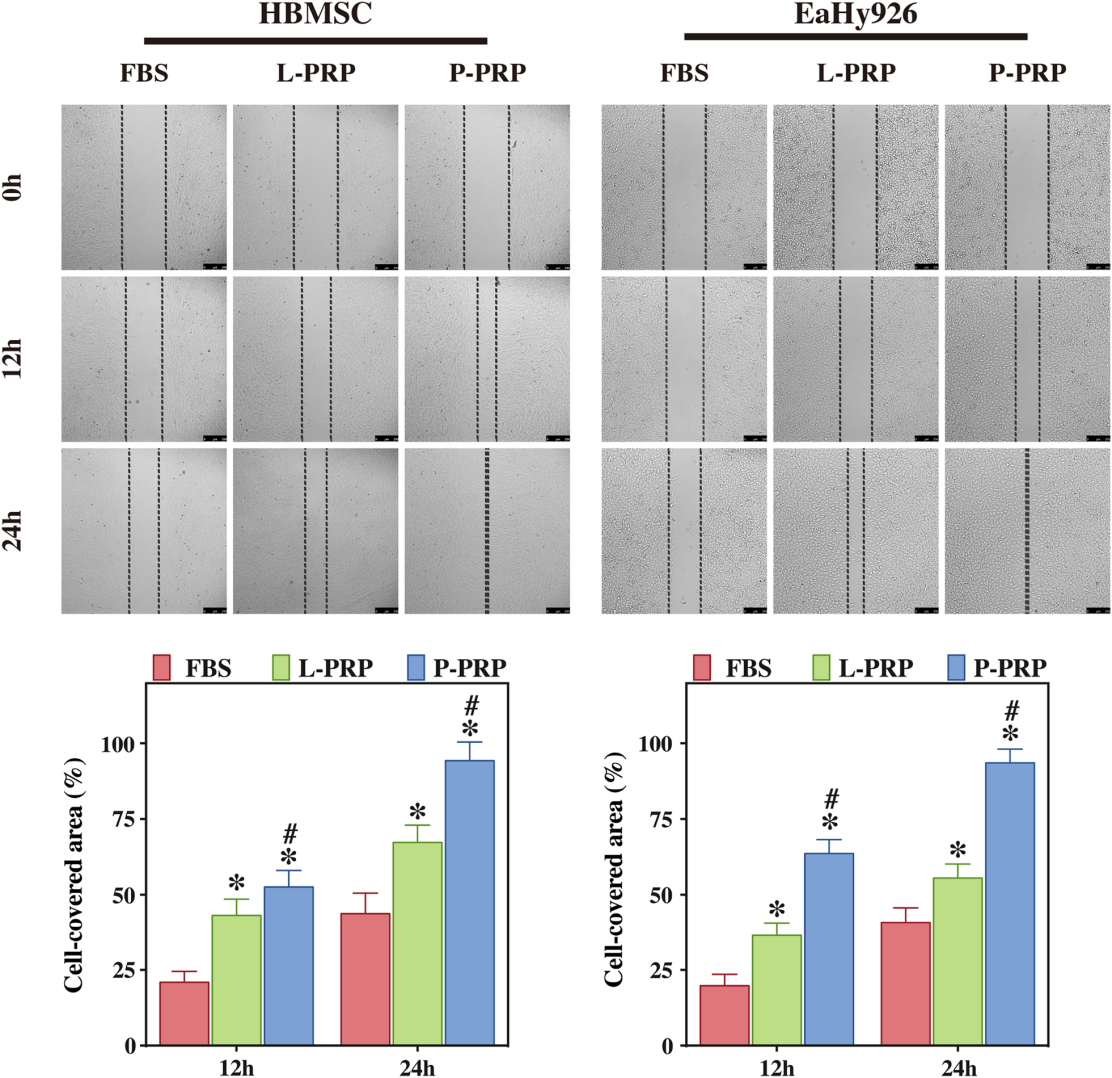
Effects of PRPs on the migration of HBMSCs and EaHy926. Cell migration was analyzed using Culture-Inserts and quantified using WimScratch software. Compared with FBS, both L-PRP and P-PRP promoted the migration of HBMSCs and EaHy926, with P-PRP showing greater effects. *Bars* represent the means and standard deviation (n = 5) and *scales* represent 250 μm; * indicates the statistically significant difference between PRPs and FBS (*P* < 0.05); ^#^ indicates the statistically significant difference between P-PRP and L-PRP (*P* < 0.05)

Cell migration analysis showed that both L-PRP and P-PRP significantly promoted the migration of HBMSCs and EaHy926 compared with FBS after incubation for 12 and 24 h, whereas P-PRP was shown to be more effective than L-PRP in aspect of the promotion of cell migration (Fig. 4).

### P-PRP promotes EaHy926 tube formation more effectively than L-PRP

**Fig. 5:**
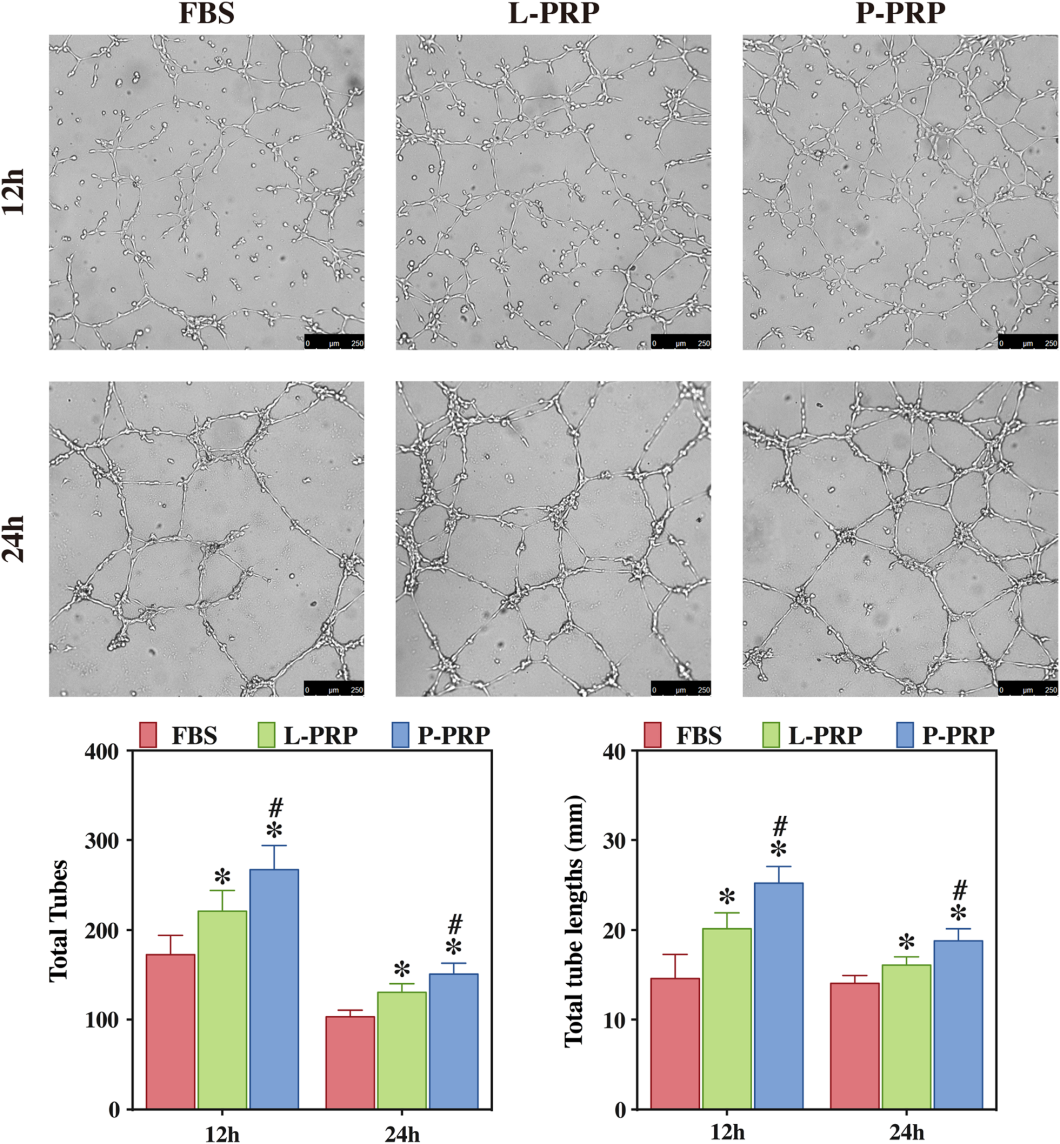
Effects of PRPs on EaHy926 tube formation. EaHy926 tube formation was analyzed using μ-slide angiogenesis plates and WimTube software quantification. Compared with FBS, both L-PRP and P-PRP promoted the tube formation of EaHy926, with P-PRP showing greater effects. *Bars* represent the means and standard deviation (n = 5) and *scales* represent 250 μm; * indicates the statistically significant difference between PRPs and FBS (*P* < 0.05); ^#^ indicates the statistically significant difference between P-PRP and L-PRP (*P* < 0.05)

The results of tube formation analysis are shown in Fig. 5. EaHy926 treated with L-PRP or P-PRP formed elongated and tube-like structures, whereas EaHy926 incubated with FBS formed incomplete or sparse tubular networks. WimTube software quantification demonstrated that both PRP treatments promoted EaHy926 tube formation in term of total tubes and total tube lengths, with P-PRP showing greater effects.

### P-PRP improves the osteogenic differentiation of HBMSCs more effectively than L-PRP

**Fig. 6:**
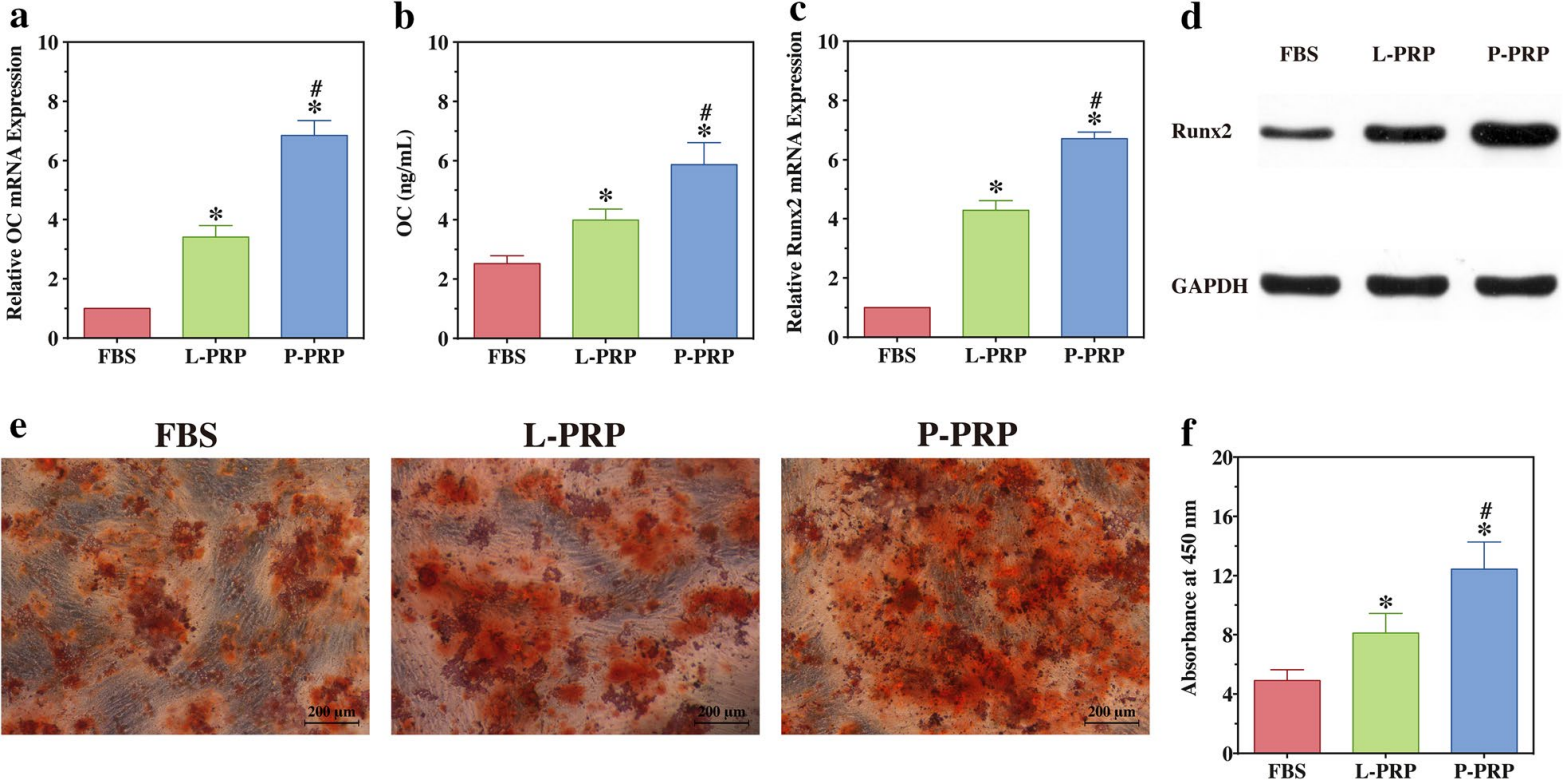
Effects of PRPs on the osteogenic differentiation of HBMSCs. **a** mRNA expression of OC of HBMSCs was detected by RT-PCR after osteogenic differentiation induction for 14 days; **b** OC concentration in conditioned medium was detected by ELISA after osteogenic differentiation induction for 14 days; **c** mRNA expression of Runx2 of HBMSCs was detected by RT-PCR after osteogenic differentiation induction for 14 days; **d** Runx2 expression was detected by western blotting after osteogenic differentiation induction for 14 days; **e** alizarin red staining of HBMSCs after osteogenic differentiation induction for 21 days; **f**, alizarin red staining was quantified by a colorimetric assay and the absorbance value was measured at 450 nm. Compared with FBS, both L-PRP and P-PRP improved the osteogenic differentiation of HBMSCs, with P-PRP showing greater effects. *Bars* represent the means and standard deviation (n = 3), and *scales* represent 200 μm; * indicates the statistically significant difference between PRPs and FBS (*P* < 0.05); ^#^ indicates the statistically significant difference between P-PRP and L-PRP (*P* < 0.05)

RT-PCR analysis revealed that mRNA expression of OC was upregulated significantly by P-PRP compared with FBS and L-PRP (*P* < 0.001), whereas it was also upregulated significantly by L-PRP compared with FBS (*P* < 0.001, Fig. 6a). Similarly, mRNA expression of Runx2 was significantly upregulated by P-PRP compared with L-PRP and FBS (*P* < 0.001), while it was also significantly increased by L-PRP compared with FBS (*P* = 0.001, Fig. 6c).

The analyses of protein expression of OC and Runx2 demonstrated that P-PRP upregulated the expression of OC (Fig. 6b) and Runx2 (Fig. 6d) significantly compared with L-PRP and FBS, while L-PRP also upregulated those compared with FBS.

Alizarin red staining showed more calcium nodules in the PRP groups compared with the FBS group, while the mineralization of HBMSCs was improved more effectively in those treated with P-PRP (Fig. 6e). The trend observed in the qualitative analysis was also shown by the results of the quantification of alizarin red staining, which demonstrated that the cultures of the P-PRP group had a significantly higher absorbance value at 450 nm compared with that of the L-PRP group, which, in turn, had a significantly higher absorbance value compared with the FBS group (Fig. 6f).

### L-PRP induces activation of NF-κB in vitro

**Fig. 7:**
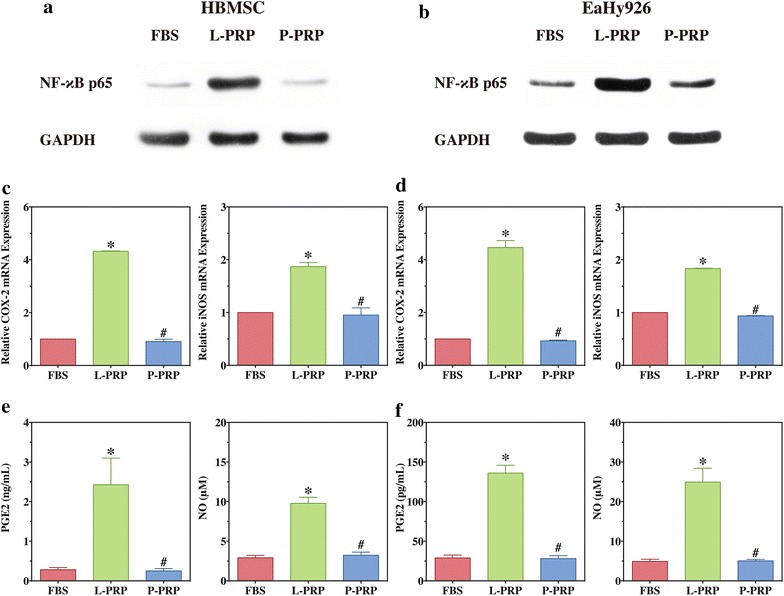
L-PRP induced activation of NF-κB in cells in vitro. Western blotting was performed to analyze expression of NF-κB p65 in the nucleus of HBMSCs (**a**) and EaHy926 (**b**); RT-PCR was conducted to detect mRNA expression of COX-2 and iNOS of HBMSCs (**c**) and EaHy926 (**d**); ELISA and Griess reaction were performed to determine PGE2 and NO production, respectively, of HBMSCs (**e**) and EaHy926 (**f**). *Bars* represent the means and standard deviation (n = 5); * indicates the statistically significant difference between PRPs and FBS (*P* < 0.05); ^#^ indicates the statistically significant difference between P-PRP and L-PRP (*P* < 0.05)

Western blotting demonstrated that NF-κB p65 was located in the cytoplasm of HBMSCs in FBS and P-PRP group (Fig. 7a), while stimulus of L-PRP resulted in the accumulation of NF-κB p65 in the nucleus (Fig. 7a). RT-PCR demonstrated that mRNA expression of COX-2 and iNOS in HBMSCs treated with L-PRP was significantly upregulated compared with HBMSCs treated with FBS or P-PRP (*P* < 0.001, Fig. 7c). Also, upregulated production of PGE2 and NO was observed in HBMSCs treated with L-PRP compared with HBMSCs treated with FBS or P-PRP (Fig. 7e).

Similarly, L-PRP treatment also resulted in the nuclear translocation of NF-κB p65 (Fig. 7b), upregulated COX-2 and iNOS mRNA expression (*P* < 0.001, Fig. 7d), and upregulated PGE2 and NO production of EaHy926 (*P* < 0.001, Fig. 7f), compared with incubation with FBS and P-PRP.

### Components of rat whole blood and PRPs

The mean leukocyte and platelet concentrations of rat whole blood were 8.10 ± 2.04 × 10^9^/L and 616.50 ± 136.248 × 10^9^/L, respectively. Similar to the components of human PRPs, an almost sixfold increase in platelet concentration was detected in both L-PRP (3735.40 ± 670.81 × 10^9^/L) and P-PRP (3800.70 ± 491.78 × 10^9^/L) compared with whole blood (*P* < 0.001). Besides that, leukocytes were concentrated in L-PRP (45.02 ± 11.01 × 10^9^/L) and almost depleted in P-PRP (0.10 ± 0.09 × 10^9^/L) compared with the whole blood (*P* < 0.001).

### P-PRP promotes the healing process of calvarial defects in rats more effectively than L-PRP

**Fig. 8:**
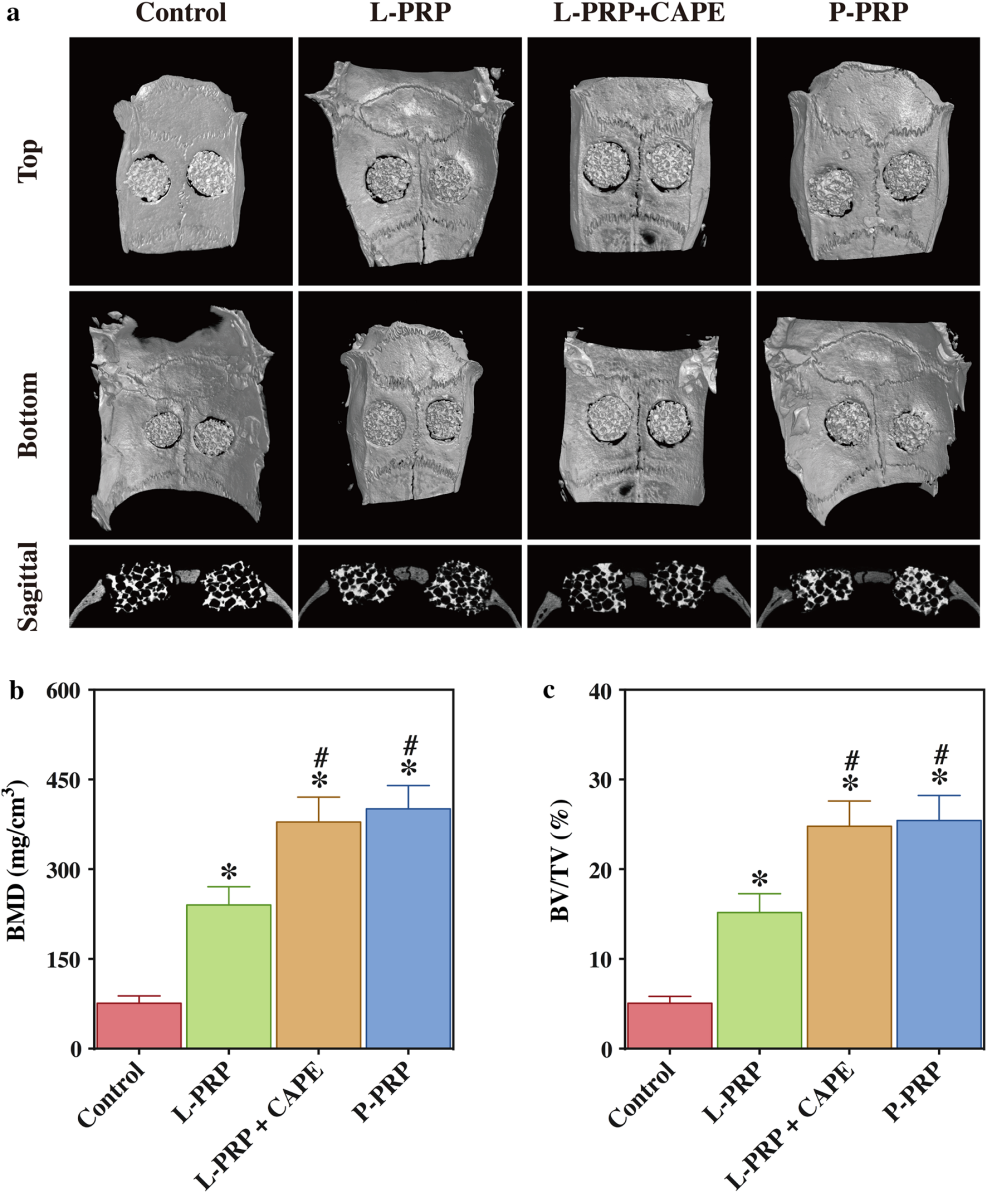
Micro-CT evaluation of bone regeneration in rat calvarial defects after 8 weeks postoperatively. **a** *Top*, *bottom* and *cross-sectional* views of the reconstructed images; **b**, **c** BMD (**b**) and BV/TV (**c**) of the regenerated bone in the defects. *Bars* represent the means and standard deviation (n = 10), and *scales* represent 200 μm; * indicates the statistically significant difference compared with the control group (*P* < 0.05); ^#^ indicates the statistically significant difference compared with the L-PRP group (*P* < 0.05)

Micro-CT showed a greater amount of newly formed bone in the defects of both PRP groups than in the defects of the control group, whereas newly formed bone in the defects of the L-PRP group appeared to be less than in the defects of the L-PRP + CAPE group and P-PRP group, which did not different from one another (Fig. 8a). Quantative analysis of micro-CT demonstrated that BMD in the P-PRP group (400.98 ± 38.84 mg/cm^3^) and L-PRP + CAPE group (378.93 ± 41.63 mg/cm^3^) were similar (*P* > 0.999), but significantly higher than that in the control group (75.90 ± 12.20 mg/cm^3^, *P* < 0.001) and L-PRP group (240.09 ± 30.69 mg/cm^3^, *P* < 0.001, Fig. 8b). A similar trend was observed in the results of BV/TV analysis (Fig. 8c), which demonstrated no significant difference between the BV/TV of the P-PRP group (25.43 ± 2.78 %) and L-PRP + CAPE group (24.79 ± 2.81 %)(*P* > 0.999), but these were higher than that of the control group (5.06 ± 0.76 %, *P* < 0.001) and L-PRP group (15.17 ± 2.10 %, *P* < 0.001, Fig. 8c).

**Fig. 9:**
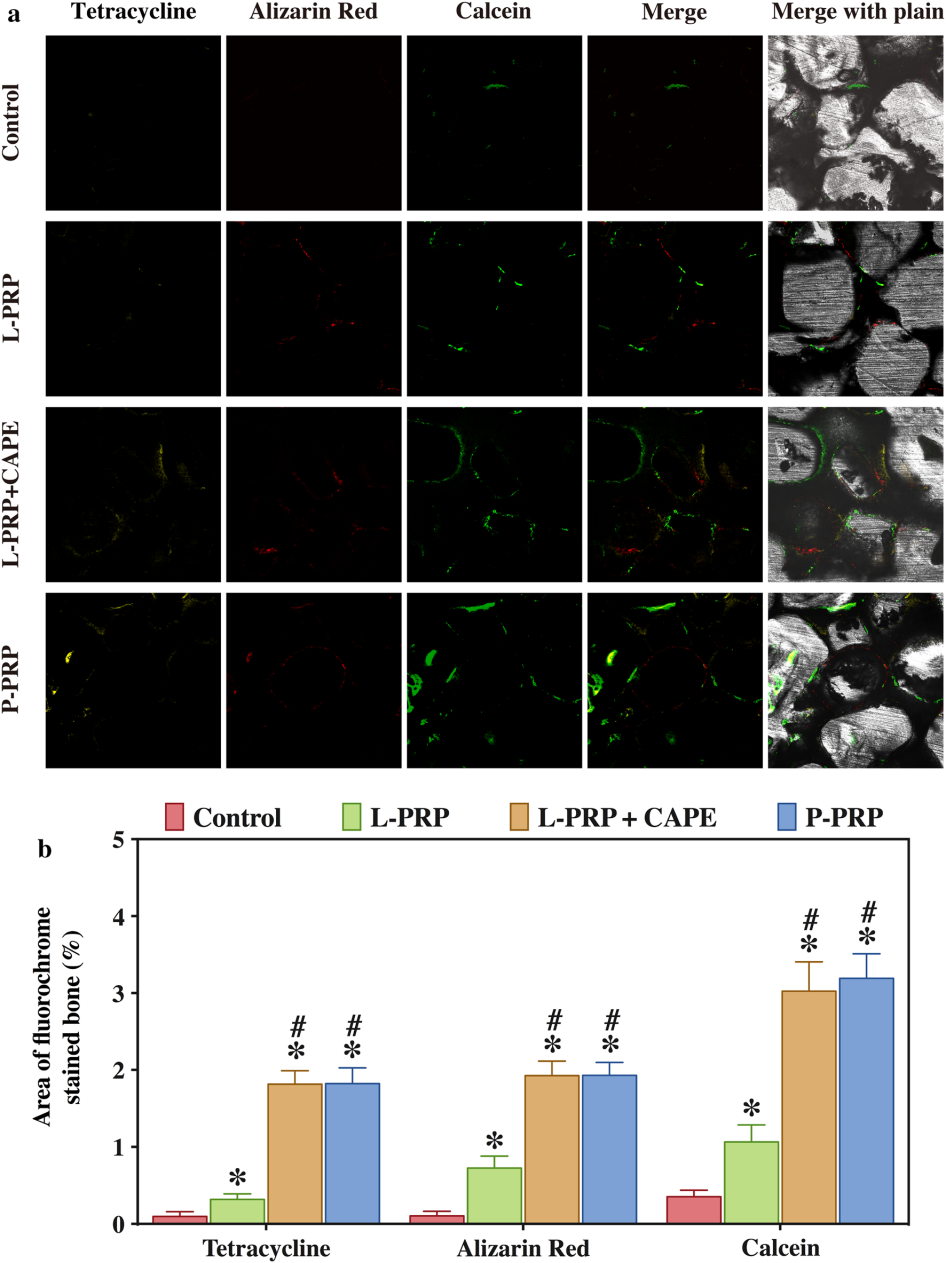
Fluorochrome-labeling of new bone formation and mineralization in the defects. **a** *Column 1* (*yellow*) shows the deposition of tetracycline at week 2, *column 2* (*red*) shows the deposition of alizarin red at week 4, *column 3* (*green*) shows the deposition of calcein at week 6, *column 4* represents the merged images of the three fluorochromes for the same group, and *column 5* represents the merged images of the three fluorochromes with a plain CLSM image for the same group; **b** the percentages of three fluorochrome areas in the defects. *Bars* represent the means and standard deviation (n = 10); * indicates the statistically significant difference compared with the control group (*P* < 0.05); ^#^ indicates the statistically significant difference compared with the L-PRP group (*P* < 0.05)

The formation and mineralization of new bone in the defects at week 2, 4, and 6 were determined histomorphometrically by the quantification of tetracycline, alizarin red and calcein fluorescence (Fig. 9). At week 2, the percentages of tetracycline labeling (yellow, Fig. 9a, column 1) in the P-PRP group (1.82 ± 0.20 %) and L-PRP + CAPE group (1.81 ± 0.18 %) were significantly greater than that in the L-PRP group (0.32 ± 0.07 %, *P* < 0.001), which, in turn, was significantly greater than that in the control group (0.10 ± 0.06 %, *P* < 0.001, Fig. 9b). Likewise, the percentages of alizarin red labeling (red, Fig. 9a, column 2) at week 4 and the calcein labeling (green, Fig. 9a, column 3), which were similar in the P-PRP group (1.93 ± 0.17 %, and 3.02 ± 0.38 %, respectively) and L-PRP + CAPE group (1.93 ± 0.19 %, and 3.02 ± 0.38 %, respectively, *P* > 0.999), lower in the L-PRP group (1.93 ± 0.19 %, and 3.02 ± 0.38 %, respectively, *P* < 0.001), and the lowest in the control group (0.10 ± 0.06 %, and 0.35 ± 0.08 %, respectively, *P* < 0.001, Fig. 9b).

**Fig. 10:**
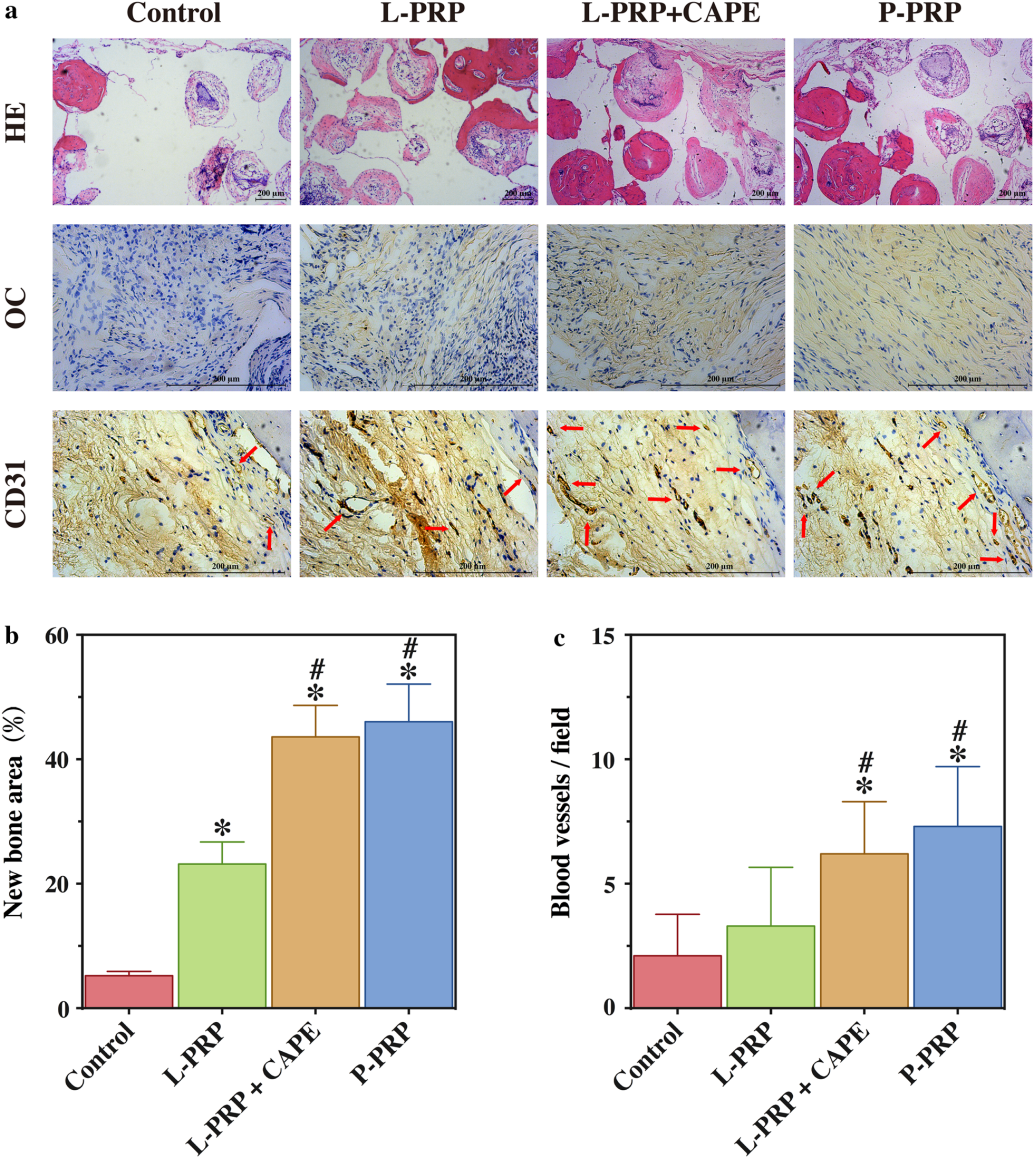
HE staining and immunohistochemical staining in the defects after 8 weeks postoperatively. **a** *line 1*, HE staining of new bone formation (*red area*) in the defects; *line 2*, the immunohistochemical staining of OC showed that there was almost no positive staining for OC in the control group, a limited amount in the L-PRP group, and a greater amount in the L-PRP + CAPE and P-PRP group; *line 3*, the immunohistochemical staining of CD31 showed that there were more new vessels, which were defined by positive CD31 staining and the typical round or oval structure (*red arrows*), in P-PRP and L-PRP + CAPE group than in the L-PRP and the control group (*line 3*); **b** quantitative analysis of the HE staining; **c** quantitative analysis of the CD31 staining. *Bars* represent the means and standard deviation (n = 10), and *scales* represent 200 μm; * indicates the statistically significant difference compared with the control group (*P* < 0.05); ^#^ indicates the statistically significant difference compared with the L-PRP group (*P* < 0.05)

HE staining was performed to evaluate new bone formation in the defects after 8 weeks postoperatively (Fig. 10a). There was abundant new bone formation in the defects of the P-PRP group and L-PRP + CAPE group, less in the defects of the L-PRP group, and little in the defects of the control group (Fig. 10a, line 1). HE staining was in accordance with the results of micro-CT and fluorochrome labeling and demonstrated that the percentages of new bone area were similar in the defects of the P-PRP group (46.05 ± 6.05 %) and L-PRP + CAPE group (43.61 ± 5.05 %, *P* > 0.999), lower in the defects of the L-PRP group (23.17 ± 3.54 %, *P* < 0.001), and the lowest in the defects of the control group (5.22 ± 0.71 %, *P* < 0.001) (Fig. 10b).

Immunohistochemical analysis was performed to evaluate the expression and distribution of OC in the defects after 8 weeks postoperatively. The results demonstrated almost no positive staining for OC in the defects of the control group, limited positive staining in the defects of the L-PRP group and a greater amount of positive staining in the defects of the P-PRP group and L-PRP + CAPE group (Fig. 10a, line 2).

Immunohistochemical staining for CD31 was performed to evaluate new blood vessel formation in the defects. Blood vessels were defined by CD31-positive staining and a typical round or oval structure. As shown in the line 3 of Fig. 10a, more blood vessels were observed in the defects of the P-PRP and L-PRP + CAPE group than L-PRP and the control group. Also, the quantitative analysis demonstrated that the number of blood vessels in the defects of the P-PRP and L-PRP + CAPE group were significantly higher than that of the L-PRP and control group (Fig. 10c).

Taken together, these results demonstrated that P-PRP had a greater capacity to promote healing process of rat calvarial defects compared with L-PRP. Furthermore, the postoperative injection of CAPE enhanced the positive effects of L-PRP on bone regeneration.

## Discussion

This study evaluated the in vitro and in vivo effects on bone regeneration of L-PRP and P-PRP, which had similar platelet and growth factor concentration but differed in leukocyte and pro-inflammatory cytokine concentration. The findings showed that in vitro, compared with L-PRP, P-PRP promoted the proliferation, viability and migration of HBMSCs and EaHy926, together with tube formation of EaHy926 and osteogenic differentiation of HBMSCs. The implantation of P-PRP preprocessed β-TCP also yielded better histological results than the implantation of L-PRP preprocessed β-TCP in vivo. Moreover, L-PRP treatment resulted in the activation of the NF-κB pathway in HBMSCs and EaHy926 in vitro while the postoperative delivery of CAPE, an inhibitor of NF-κB activation, enhanced the histological results of the implantation of L-PRP preprocessed β-TCP in vivo.

Since Whitman advocated for the first time that PRP might be a potent source of autologous growth factors in 1997 [45], efforts have been devoted to evaluate the effects of PRP on bone regeneration. Numerous animal models have demonstrated the positive effects of PRP on promoting bone regeneration in calvarial defects [46, 47], mandibular defects [48, 49], tibia defects [18, 50] and femoral defects [51, 52]. Moreover, clinical studies implied that the use of PRP might represent a promising alterative for autologous bone graft in the treatment of bone defects [53] and nonunion [54], as well as in distraction osteogenesis [55]. As a result, PRP has attracted increasing clinical interest in the field of orthopaedic surgery, as well as many other medical fields, and the market for PRP is expected to worth $126 millions by 2016 [7].

The rationale behind the PRP therapy arises from the growth factors released from platelet α-granules, and therefore, PRP is usually activated before application for degranulation of platelets. Bovine thrombin is often used to activate platelets, causing them to release 70 % of stored growth factors within 10 min and nearly 100 % within 1 h [56]. However, exogenous bovine thrombin may lead to complications associated with the formation of antibodies against human coagulation proteins, and result in an immune-mediated coagulopathy [57]. Moreover, bovine thrombin might impair the osteoconductivity of materials used in orthopaedic surgery [58]. To obviate the risks associated with thrombin, methods to activate PRP that do not rely on thrombin have been explored. CaCl_2_ is a well-accepted platelet activator and has been used extensively [29, 30, 59]. It has been demonstrated that addition of CaCl_2_ generates the clotting mechanism to activate the platelets in PRP and stimulates the formation of native thrombin to mimick the physiological clotting process and enable a more sustained release of growth factors [60, 61]. As a result, CaCl_2_ activation has also gained popularity in clinical practice [26], and we also used CaCl2 to activate PRP formulations in this study. An alternative activator recently reported is type I collagen. Fufa et al. reported that activation with type I collagen was equally effective as thrombin in stimulating the release of PDGF-AB and VEGF [62]. In the further study by the same group, Harrison et al. reported that collagen might be more effective than thrombin activation, as thrombin activated platelets to release growth factors immediately while collagen resulted in a gradual accumulation of growth factors [63]. As type I collagen is the most abundant protein found in humans and is likely to be exposed in the environment of PRP application, further study is needed to determine whether preactivation of PRP is necessary.

PDGF-AB, TGF-β1, and VEGF have been detected consistently in PRP [7, 8]. PDGF-AB is a potent chemokine and regulator of cell proliferation and extracellular matrix deposition [15, 64] while TGF-β1 is involved in cell proliferation and apoptosis, as well as extracellular matrix deposition [65, 66]. Moreover, PDGF-AB and TGF-β1 have been shown to promote bone regeneration through enhanced osteogenesis [9, 10]. VEGF is an important stimulus for angiogenesis, which is important for new bone formation [12, 67]. Although IGF has also been detected consistently in PRP and demonstrated to have beneficial effects on cell proliferation and bone matrix synthesis [66], exercise and nutritional status, which are hard to control in volunteers, may affect IGF concentration in the whole blood and therefore in PRP [68]. Therefore, IGF was excluded from the analysis, and PDGF-AB, TGF-β1, and VEGF concentrations were selected to characterize L-PRP and P-PRP used in the current study together with platelet concentration. Our findings showed that the L-PRP and P-PRP used in the current study contained similar concentrations of platelets and growth factors. Additionally, we found positive correlations between platelet and growth factors concentration. These findings imply that it is platelets, rather than leukocytes, that are the major source of growth factors in PRP, and leukocyte-depletion may have no influence on the presence of concentration of growth factors in PRP, which is believed to be the basis of PRP therapy.

Interestingly, the in vitro and in vivo studies revealed that the similar levels of platelets and growth factors in L-PRP and P-PRP did not result in similar therapeutic effects and P-PRP was shown to be more effective than L-PRP. The possible reason for this phenomenon may be the concentration of leukocytes and pro-inflammatory cytokines. Our findings support other studies demonstrating that leukocytes in L-PRP provide increased IL-1β and TNF-α [69]. IL-1β and TNF-α are recognised as primary cytokines for inflammation and the effects of inflammation on bone regeneration are biphasic. Although an inflammatory response is required to initiate bone regeneration, excessive inflammation delays or inhibits it through inhibited osteogenesis and enhanced osteoclastogenesis [70, 71]. Also, inordinate inflammation may facilitate adipogenesis to impair osteogenesis [72] and in addition, may also lead to endothelial dysfunction to inhibit the proliferation, viability, migration, and angiogenesis of endothelial cells [73–75]. Studies demonstrating that anti-inflammatory drugs enhance bone regeneration also provide substantial proof for the harmful effects of excessive inflammation on bone regeneration [76, 77]. Hence, the increased pro-inflammatory cytokines released from the leukocytes in L-PRP may induce an inflammatory environment which overwhelms or counters the beneficial effects of the growth factors resulting in the inferior effects on bone regeneration observed here of L-PRP compared with P-PRP.

The NF-κB pathway is intimately involved in the regulation of inflammatory responses. Activation of the NF-κB pathway has been shown to be responsible for the harmful effects of IL-1β and TNF-α on tissue regeneration [72, 73, 75, 78, 79], however, the effects of increased levels of these cytokines in L-PRP on NF-κB have not been evaluated. IL-1β and TNF-α activate NF-κB pathway via the canonical pathway which involves the nuclear translocation of NF-κB heterodimers [80, 81]. The translocated NF-κB heterodimers then upregulate the expression of downstream inflammation-related genes (COX-2 and iNOS) and the synthesis of their product (PGE2 and NO). Similar to the biphasic effects of inflammation on bone regeneration, regulated PGE2 and NO production are also needed in the bone regeneration, possibly because that PGE2 and NO may be required in the immunosuppression by MSCs, which may have beneficial effects on tissue repair [82], and excessive production of PGE2 and NO may have harmful effects on bone regeneration through inhibited osteogenesis and angiogenesis [72, 73, 75, 83]. Therefore, protein expression of NF-κB p65 in the nucleus, mRNA expression of COX-2 and iNOS, and production of PGE2 and NO were analyzed to evaluate the effects of PRP treatments on the NF-κB pathway. We found that L-PRP treatment induced the activation of NF-κB in HBMSCs and EaHy926 through the canonical pathway, upregulated COX-2 and iNOS mRNA expression, production of PGE2 and NO. Moreover, the in vivo studies showed that the use of CAPE, a specific inhibitor of NF-κB activation, improved the in vivo effects of L-PRP treatment on bone regeneration. Hence, the activation of NF-κB pathway may play a role in the inferior effects of L-PRP compared with P-PRP on bone regeneration.

In clinical practice, the inferior effects of L-PRP to P-PRP are also reflected in post-injection reactions. A randomized control trial comparing the effects of L-PRP and P-PRP on osteoarthritis demonstrated that L-PRP produced more pain and swelling reaction than P-PRP [26]. It is well established that excessive inflammation is involved in pain reaction [84]. Also, inhibitors of COX-2 have been used widely in pain relief [85] and the inhibition of NF-κB activation has been proved to be a novel drug target for pain relief [86]. Hence, NF-κB activation may also play a role in the increased incidence of pain reaction after L-PRP treatment and the use of P-PRP in the treatment of bone defects may not only represent a more effective approach to promote bone regeneration, but also represent a safer approach to avoid adverse events compared with the use of L-PRP.

The major limitation of this study is that the rat calvarial defects model used does not allow the assessment of the biological response of the implanted material to a physiological biomechanical loading, which may influence bone regeneration strongly [87]. Hence, further studies using bone defects models on anatomical load-bearing locations, such as mandible, distal femur and proximal tibia, of larger animals are needed to evaluate the findings of the current study.

## Conclusions

Although L-PRP promoted bone regeneration by the enhanced proliferation, viability, migration of cells in vitro and angiogenesis and osteogenesis in vitro and in vivo, leukocytes in L-PRP may produce harmful effects through the activation of NF-κB and lead to the inferior effects of L-PRP compared with P-PRP. Therefore, P-PRP may be more suitable for bone regeneration, and the combined use of P-PRP and β-TCP may represent a safe, simple, and effective alternative option for autogenous bone graft in the treatment of bone defects.

## Abbreviations

β-TCP: β-tricalcium phosphate
PRP: platelet-rich plasma
PDGF: platelet-derived growth factor
TGF-β1: transforming growth factor β1
VEGF: vascular endothelial growth factor
IGF: insulin-like growth factor
IL-1β: interleukin-1β
TNF-α: tumor necrosis factor-α
NF-κB: nuclear factor κB
IκB: inhibitory κB
L-PRP: leukocyte- and platelet-rich plasma
P-PRP: pure platelet-rich plasma
ACD-A: acid-citrate dextrose solution A
ELISA: enzyme-linked immunosorbent assay
HBMSCs: human bone marrow-derived mesenchymal stem cells
α-MEM: α-modification of minimum essential medium
FBS: fetal bovine serum
DMEM: Dulbecco’s modified eagle’s medium
CCK-8: cell counting kit-8
EDTA: ethylenediamine-tetraacetic acid
mRNA: message RNA
RT-PCR: real-time quantitative polymerase chain reaction
Runx2: runt-related transcription factor 2
OC: osteocalcin
iNOS: inducible nitric oxide synthase
COX-2: cyclooxygenase-2
PGE2: prostaglandin E2
NO: nitric oxide
CAPE: caffeic acid phenethyl ester
CT: computed tomography
BMD: bone mineral density
BV/TV: bone volume to total bone volume
HE: hematoxylin and eosin
SD: standard deviation
ANOVA: one-way analysis of variance

## References

[1] Dimitriou R, Jones E, McGonagle D, Giannoudis PV (2011). Bone regeneration: current concepts and future directions. BMC Med.

[2] Calori GM, Colombo M, Mazza EL, Mazzola S, Malagoli E, Mineo GV (2014). Incidence of donor site morbidity following harvesting from iliac crest or RIA graft. Injury.

[3] Oryan A, Alidadi S, Moshiri A, Maffulli N (2014). Bone regenerative medicine: classic options, novel strategies, and future directions. J Orthop Surg Res.

[4] Yu H, VandeVord PJ, Mao L, Matthew HW, Wooley PH, Yang SY (2009). Improved tissue-engineered bone regeneration by endothelial cell mediated vascularization. Biomaterials.

[5] Hirota M, Matsui Y, Mizuki N, Kishi T, Watanuki K, Ozawa T (2009). Combination with allogenic bone reduces early absorption of beta-tricalcium phosphate (beta-TCP) and enhances the role as a bone regeneration scaffold. Experimental animal study in rat mandibular bone defects. Dent Mater J.

[6] Castillo TN, Pouliot MA, Kim HJ, Dragoo JL (2011). Comparison of growth factor and platelet concentration from commercial platelet-rich plasma separation systems. Am J Sports Med.

[7] Magalon J, Bausset O, Serratrice N, Giraudo L, Aboudou H, Veran J (2014). Characterization and comparison of 5 platelet-rich plasma preparations in a single-donor model. Arthroscopy.

[8] Mazzocca AD, McCarthy MB, Chowaniec DM, Cote MP, Romeo AA, Bradley JP (2012). Platelet-rich plasma differs according to preparation method and human variability. J Bone Joint Surg Am.

[9] Joyce ME, Roberts AB, Sporn MB, Bolander ME (1990). Transforming growth factor-beta and the initiation of chondrogenesis and osteogenesis in the rat femur. J Cell Biol.

[10] Centrella M, Massague J, Canalis E (1986). Human platelet-derived transforming growth factor-beta stimulates parameters of bone growth in fetal rat calvariae. Endocrinology.

[11] Hock JM, Centrella M, Canalis E (1988). Insulin-like growth factor I has independent effects on bone matrix formation and cell replication. Endocrinology.

[12] Kanczler JM, Oreffo RO (2008). Osteogenesis and angiogenesis: the potential for engineering bone. Eur Cell Mater.

[13] Wozney JM, Rosen V, Celeste AJ, Mitsock LM, Whitters MJ, Kriz RW (1988). Novel regulators of bone formation: molecular clones and activities. Science.

[14] Roubelakis MG, Trohatou O, Roubelakis A, Mili E, Kalaitzopoulos I, Papazoglou G (2014). Platelet-rich plasma (PRP) promotes fetal mesenchymal stem/stromal cell migration and wound healing process. Stem Cell Rev.

[15] Xie X, Wang Y, Zhao C, Guo S, Liu S, Jia W (2012). Comparative evaluation of MSCs from bone marrow and adipose tissue seeded in PRP-derived scaffold for cartilage regeneration. Biomaterials.

[16] Schar MO, Diaz-Romero J, Kohl S, Zumstein MA, Nesic D (2015). Platelet-rich concentrates differentially release growth factors and induce cell migration in vitro. Clin Orthop Relat Res.

[17] Cho K, Kim JM, Kim MH, Kang SS, Kim G, Choi SH (2013). Scintigraphic evaluation of osseointegrative response around calcium phosphate-coated titanium implants in tibia bone: effect of platelet-rich plasma on bone healing in dogs. Eur Surg Res.

[18] Hakimi M, Grassmann JP, Betsch M, Schneppendahl J, Gehrmann S, Hakimi AR (2014). The composite of bone marrow concentrate and PRP as an alternative to autologous bone grafting. PLoS One.

[19] Messora MR, Nagata MJ, Pola NM, de Campos N, Fucini SE, Furlaneto FA (2013). Effect of platelet-rich plasma on bone healing of fresh frozen bone allograft in mandibular defects: a histomorphometric study in dogs. Clin Oral Implants Res.

[20] Mooren RE, Hendriks EJ, van den Beucken JJ, Merkx MA, Meijer GJ, Jansen JA (2010). The effect of platelet-rich plasma in vitro on primary cells: rat osteoblast-like cells and human endothelial cells. Tissue Eng Part A.

[21] Kakudo N, Morimoto N, Kushida S, Ogawa T, Kusumoto K (2014). Platelet-rich plasma releasate promotes angiogenesis in vitro and in vivo. Med Mol Morphol.

[22] Zhong D, Wang CG, Yin K, Liao Q, Zhou X, Liu AS (2014). In vivo ossification of a scaffold combining beta-tricalcium phosphate and platelet-rich plasma. Exp Ther Med.

[23] Okamoto S, Ikeda T, Sawamura K, Nagae M, Hase H, Mikami Y (2012). Positive effect on bone fusion by the combination of platelet-rich plasma and a gelatin beta-tricalcium phosphate sponge: a study using a posterolateral fusion model of lumbar vertebrae in rats. Tissue Eng Part A.

[24] Intini G, Andreana S, Intini FE, Buhite RJ, Bobek LA (2007). Calcium sulfate and platelet-rich plasma make a novel osteoinductive biomaterial for bone regeneration. J Transl Med.

[25] McCarrel TM, Minas T, Fortier LA (2012). Optimization of leukocyte concentration in platelet-rich plasma for the treatment of tendinopathy. J Bone Joint Surg Am.

[26] Filardo G, Kon E, Pereira Ruiz MT, Vaccaro F, Guitaldi R, Di Martino A (2012). Platelet-rich plasma intra-articular injections for cartilage degeneration and osteoarthritis: single- versus double-spinning approach. Knee Surg Sports Traumatol Arthrosc.

[27] Riboh JC, Saltzman BM, Yanke AB, Fortier L, Cole BJ. Effect of leukocyte concentration on the efficacy of platelet-rich plasma in the treatment of knee osteoarthritis. Am J Sports Med. 2015. 10.1177/0363546515580787 25925602

[28] Hayden MS, Ghosh S (2008). Shared principles in NF-kappaB signaling. Cell.

[29] Bausset O, Giraudo L, Veran J, Magalon J, Coudreuse JM, Magalon G (2012). Formulation and storage of platelet-rich plasma homemade product. Biores Open Access.

[30] Cavallo C, Filardo G, Mariani E, Kon E, Marcacci M, Pereira Ruiz MT (2014). Comparison of platelet-rich plasma formulations for cartilage healing: an in vitro study. J Bone Joint Surg Am.

[31] Assirelli E, Filardo G, Mariani E, Kon E, Roffi A, Vaccaro F (2015). Effect of two different preparations of platelet-rich plasma on synoviocytes. Knee Surg Sports Traumatol Arthrosc.

[32] Braun HJ, Kim HJ, Chu CR, Dragoo JL (2014). The effect of platelet-rich plasma formulations and blood products on human synoviocytes: implications for intra-articular injury and therapy. Am J Sports Med.

[33] Zhou Y, Zhang J, Wu H, Hogan MV, Wang JH (2015). The differential effects of leukocyte-containing and pure platelet-rich plasma (PRP) on tendon stem/progenitor cells—implications of PRP application for the clinical treatment of tendon injuries. Stem Cell Res Ther.

[34] Cross JA, Cole BJ, Spatny KP, Sundman E, Romeo AA, Nicholson GP et al. Leukocyte-reduced platelet-rich plasma normalizes matrix metabolism in torn human rotator cuff tendons. Am J Sports Med. 2015. 10.1177/0363546515608157 26460099

[35] McCarrel T, Fortier L (2009). Temporal growth factor release from platelet-rich plasma, trehalose lyophilized platelets, and bone marrow aspirate and their effect on tendon and ligament gene expression. J Orthop Res.

[36] Pifer MA, Maerz T, Baker KC, Anderson K (2014). Matrix metalloproteinase content and activity in low-platelet, low-leukocyte and high-platelet, high-leukocyte platelet rich plasma (PRP) and the biologic response to PRP by human ligament fibroblasts. Am J Sports Med.

[37] Zhu Z, Yin J, Guan J, Hu B, Niu X, Jin D (2014). Lithium stimulates human bone marrow derived mesenchymal stem cell proliferation through GSK-3beta-dependent beta-catenin/Wnt pathway activation. FEBS J.

[38] Livak KJ, Schmittgen TD (2001). Analysis of relative gene expression data using real-time quantitative PCR and the 2(-Delta Delta C(T)) method. Methods.

[39] Lee AJ, Hodges S, Eastell R (2000). Measurement of osteocalcin. Ann Clin Biochem.

[40] Ding H, Gao YS, Wang Y, Hu C, Sun Y, Zhang C (2014). Dimethyloxaloylglycine increases the bone healing capacity of adipose-derived stem cells by promoting osteogenic differentiation and angiogenic potential. Stem Cells Dev.

[41] Zhao WX, Wang L, Yang JL, Li LZ, Xu WM, Li T (2014). Caffeic acid phenethyl ester attenuates pro-inflammatory and fibrogenic phenotypes of LPS-stimulated hepatic stellate cells through the inhibition of NF-kappaB signaling. Int J Mol Med.

[42] Almasry SM, Soliman HM, El-Tarhouny SA, Algaidi SA, Ragab EM (2015). Platelet rich plasma enhances the immunohistochemical expression of platelet derived growth factor and vascular endothelial growth factor in the synovium of the meniscectomized rat models of osteoarthritis. Ann Anat.

[43] Xia L, Lin K, Jiang X, Fang B, Xu Y, Liu J (2014). Effect of nano-structured bioceramic surface on osteogenic differentiation of adipose derived stem cells. Biomaterials.

[44] Gunay A, Arpag OF, Atilgan S, Yaman F, Atalay Y, Acikan I (2014). Effects of caffeic acid phenethyl ester on palatal mucosal defects and tooth extraction sockets. Drug Des Devel Ther.

[45] Whitman DH, Berry RL, Green DM (1997). Platelet gel: an autologous alternative to fibrin glue with applications in oral and maxillofacial surgery. J Oral Maxillofac Surg.

[46] Messora MR, Nagata MJ, Mariano RC, Dornelles RC, Bomfim SR, Fucini SE (2008). Bone healing in critical-size defects treated with platelet-rich plasma: a histologic and histometric study in rat calvaria. J Periodontal Res.

[47] Aghaloo TL, Moy PK, Freymiller EG (2002). Investigation of platelet-rich plasma in rabbit cranial defects: a pilot study. J Oral Maxillofac Surg.

[48] Messora MR, Nagata MJ, Fucini SE, Pola NM, Campos N, de Oliveira GC (2014). Effect of platelet-rich plasma on the healing of mandibular defects treated with fresh frozen bone allograft: a radiographic study in dogs. J Oral Implantol.

[49] Pieri F, Lucarelli E, Corinaldesi G, Fini M, Aldini NN, Giardino R (2009). Effect of mesenchymal stem cells and platelet-rich plasma on the healing of standardized bone defects in the alveolar ridge: a comparative histomorphometric study in minipigs. J Oral Maxillofac Surg.

[50] Nather A, Wong KL, David V, Pereira BP (2012). Allografts with autogenous platelet-rich plasma for tibial defect reconstruction: a rabbit study. J Orthop Surg (Hong Kong).

[51] Rai B, Oest ME, Dupont KM, Ho KH, Teoh SH, Guldberg RE (2007). Combination of platelet-rich plasma with polycaprolactone-tricalcium phosphate scaffolds for segmental bone defect repair. J Biomed Mater Res A.

[52] Nair MB, Varma HK, Menon KV, Shenoy SJ, John A (2009). Reconstruction of goat femur segmental defects using triphasic ceramic-coated hydroxyapatite in combination with autologous cells and platelet-rich plasma. Acta Biomater.

[53] Gubina B, Rozman P, Biscevic M, Domanovic D, Smrke D (2014). The influence of allogeneic platelet gel on the morphology of human long bones. Coll Antropol.

[54] Memeo A, Verdoni F, De Bartolomeo O, Albisetti W, Pedretti L (2014). A new way to treat forearm post-traumatic non-union in young patients with intramedullary nailing and platelet-rich plasma. Injury.

[55] Lee DH, Ryu KJ, Kim JW, Kang KC, Choi YR (2014). Bone marrow aspirate concentrate and platelet-rich plasma enhanced bone healing in distraction osteogenesis of the tibia. Clin Orthop Relat Res.

[56] Marx RE (2001). Platelet-rich plasma (PRP): what is PRP and what is not PRP?. Implant Dent.

[57] Lawson JH (2006). The clinical use and immunologic impact of thrombin in surgery. Semin Thromb Hemost.

[58] Han B, Woodell-May J, Ponticiello M, Yang Z, Nimni M (2009). The effect of thrombin activation of platelet-rich plasma on demineralized bone matrix osteoinductivity. J Bone Joint Surg Am.

[59] Liu J, Song W, Yuan T, Xu Z, Jia W, Zhang C (2014). A comparison between platelet-rich plasma (PRP) and hyaluronate acid on the healing of cartilage defects. PLoS One.

[60] Anitua E, Sanchez M, Nurden AT, Nurden P, Orive G, Andia I (2006). New insights into and novel applications for platelet-rich fibrin therapies. Trends Biotechnol.

[61] Anitua E, Zalduendo MM, Alkhraisat MH, Orive G (2013). Release kinetics of platelet-derived and plasma-derived growth factors from autologous plasma rich in growth factors. Ann Anat.

[62] Fufa D, Shealy B, Jacobson M, Kevy S, Murray MM (2008). Activation of platelet-rich plasma using soluble type I collagen. J Oral Maxillofac Surg.

[63] Harrison S, Vavken P, Kevy S, Jacobson M, Zurakowski D, Murray MM (2011). Platelet activation by collagen provides sustained release of anabolic cytokines. Am J Sports Med.

[64] Heldin CH, Eriksson U, Ostman A (2002). New members of the platelet-derived growth factor family of mitogens. Arch Biochem Biophys.

[65] Blobe GC, Schiemann WP, Lodish HF (2000). Role of transforming growth factor beta in human disease. N Engl J Med.

[66] Foster TE, Puskas BL, Mandelbaum BR, Gerhardt MB, Rodeo SA (2009). Platelet-rich plasma: from basic science to clinical applications. Am J Sports Med.

[67] Zelzer E, McLean W, Ng YS, Fukai N, Reginato AM, Lovejoy S (2002). Skeletal defects in VEGF(120/120) mice reveal multiple roles for VEGF in skeletogenesis. Development.

[68] Berg U, Gustafsson T, Sundberg CJ, Carlsson-Skwirut C, Hall K, Jakeman P (2006). Local changes in the insulin-like growth factor system in human skeletal muscle assessed by microdialysis and arterio-venous differences technique. Growth Horm IGF Res.

[69] Sundman EA, Cole BJ, Fortier LA (2011). Growth factor and catabolic cytokine concentrations are influenced by the cellular composition of platelet-rich plasma. Am J Sports Med.

[70] Hardy R, Cooper MS (2009). Bone loss in inflammatory disorders. J Endocrinol.

[71] Lacey DC, Simmons PJ, Graves SE, Hamilton JA (2009). Proinflammatory cytokines inhibit osteogenic differentiation from stem cells: implications for bone repair during inflammation. Osteoarthritis Cartilage.

[72] Noack C, Hempel U, Preissler C, Dieter P (2015). Prostaglandin E2 impairs osteogenic and facilitates adipogenic differentiation of human bone marrow stromal cells. Prostaglandins Leukot Essent Fatty Acids.

[73] Lee KS, Kim J, Kwak SN, Lee KS, Lee DK, Ha KS (2014). Functional role of NF-kappaB in expression of human endothelial nitric oxide synthase. Biochem Biophys Res Commun.

[74] Roubille F, Busseuil D, Shi Y, Nachar W, Mihalache-Avram T, Mecteau M (2014). The interleukin-1beta modulator gevokizumab reduces neointimal proliferation and improves reendothelialization in a rat carotid denudation model. Atherosclerosis.

[75] Chang Y, Chang TC, Lee JJ, Chang NC, Huang YK, Choy CS (2014). Sanguis draconis, a dragon’s blood resin, attenuates high glucose-induced oxidative stress and endothelial dysfunction in human umbilical vein endothelial cells. ScientificWorldJournal..

[76] Ratanavaraporn J, Furuya H, Tabata Y (2012). Local suppression of pro-inflammatory cytokines and the effects in BMP-2-induced bone regeneration. Biomaterials.

[77] Tan Y, Montgomery SR, Aghdasi BG, Inoue H, Kaner T, Tian H (2013). The effect of corticosteroid administration on soft-tissue inflammation associated with rhBMP-2 use in a rodent model of inflammation. Spine.

[78] Montiel-Davalos A, de Ibarra-Sanchez MJ, Ventura-Gallegos JL, Alfaro-Moreno E, Lopez-Marure R (2010). Oxidative stress and apoptosis are induced in human endothelial cells exposed to urban particulate matter. Toxicol In Vitro.

[79] Huang RL, Yuan Y, Tu J, Zou GM, Li Q (2014). Exaggerated inflammatory environment decreases BMP-2/ACS-induced ectopic bone mass in a rat model: implications for clinical use of BMP-2. Osteoarthritis Cartilage.

[80] Ledoux AC, Perkins ND (2014). NF-kappaB and the cell cycle. Biochem Soc Trans.

[81] Marcu KB, Otero M, Olivotto E, Borzi RM, Goldring MB (2010). NF-κB signaling: multiple angles to target OA. Curr Drug Targets.

[82] Shi Y, Hu G, Su J, Li W, Chen Q, Shou P (2010). Mesenchymal stem cells: a new strategy for immunosuppression and tissue repair. Cell Res.

[83] Armour KJ, Armour KE, van’t Hof RJ, Reid DM, Wei XQ, Liew FY (2001). Activation of the inducible nitric oxide synthase pathway contributes to inflammation-induced osteoporosis by suppressing bone formation and causing osteoblast apoptosis. Arthritis Rheum.

[84] Millan MJ (1999). The induction of pain: an integrative review. Prog Neurobiol.

[85] FitzGerald GA, Patrono C (2001). The coxibs, selective inhibitors of cyclooxygenase-2. N Engl J Med.

[86] Tegeder I, Niederberger E, Schmidt R, Kunz S, Guhring H, Ritzeler O (2004). Specific Inhibition of IkappaB kinase reduces hyperalgesia in inflammatory and neuropathic pain models in rats. J Neurosci.

[87] Carter DR, Beaupre GS, Giori NJ, Helms JA (1998). Mechanobiology of skeletal regeneration. Clin Orthop Relat Res.

